# Lifestyle Interventions for Polycystic Ovary Syndrome: Cross-Sectional Survey to Assess Women's Treatment and Outcome Preferences

**DOI:** 10.2196/17126

**Published:** 2020-09-02

**Authors:** Laura R Saslow, James E Aikens

**Affiliations:** 1 Department of Health Behavior and Biological Sciences School of Nursing University of Michigan Ann Arbor, MI United States; 2 Department of Family Medicine University of Michigan Ann Arbor, MI United States

**Keywords:** polycystic ovary syndrome, lifestyle intervention, online and mobile, health psychology, nutrition

## Abstract

**Background:**

Polycystic ovary syndrome (PCOS) is the most common endocrine disorder in women of reproductive age. Diet and lifestyle programs improve health, but women’s preferences for these programs have not been formally explored.

**Objective:**

The aim of our study was to examine diet and lifestyle program preferences among women with PCOS.

**Methods:**

We conducted a cross-sectional online survey of women with PCOS.

**Results:**

At least half of the 197 respondents expressed strong interest in programs addressing energy level, anxiety, depression, weight, diabetes prevention, menstrual period regulation, and hirsutism. Similarly, at least half reported willingness to modify their sleep, stress, and physical activity; and slightly less than half reported willingness to adopt a very low-carbohydrate, paleo, or low–glycemic index diet. At least half reported interest in online or mobile programs and email-based mentoring. Younger age was associated with interest in help with acne and fertility; higher body mass index was associated with wanting help with weight loss, energy, and anxiety; and greater stress eating was associated with wanting help with depression, anxiety, and menstrual period regulation.

**Conclusions:**

To our knowledge, this is the first study to examine attitudes and preferences of women with PCOS toward such programs. Future online and mobile diet and lifestyle programs may be able to capitalize on this information to better target this population’s expressed preferences.

## Introduction

Polycystic ovary syndrome (PCOS) is the most common endocrine disorder in women of reproductive age. Women with it often experience obesity, insulin resistance, hyperinsulinemia, infertility, and clinical manifestations of hyperandrogenism (chronic anovulation, hirsutism, and acne) as well as have a higher risk for cardiovascular disease and type 2 diabetes [1,2].

Women with PCOS can face other challenges. On average, they experience higher levels of depressive [3] and anxiety symptoms [4], which, when elevated, may increase their use of maladaptive coping strategies such as escape–avoidance coping (wishful thinking or behavior to avoid a problem) and lower levels of adaptive coping such as planful problem solving and positive reappraisal (focusing on positive meaning and growth) [5]. They may feel abnormal and less feminine because many of their symptoms, such as hirsutism and infertility, are not traditionally emblematic of femininity [6] and because the popular press often depicts them as less able to fulfill their roles as wives and mothers [7]. On the other hand, online discussion boards and in-person support groups may be able to provide them socioemotional and informational social support [8,9].

The Androgen Excess and PCOS Society calls for the primary treatment of metabolic complications for women with PCOS to be through diet and lifestyle programs [10,11]. Similarly, the Endocrine Society encourages these interventions for overweight or obese women with PCOS [12]. Thus, diet and lifestyle programs should be playing a central role in treating women with PCOS. However, previous trials of PCOS diet and lifestyle interventions have had high dropout rates [13-15].

These high dropout rates [13-15] have been related to various factors. For example, participants who dropped out had higher baseline testosterone [16], insulin resistance [14], or weight [14] than those of participants who completed the intervention. Moreover, women with PCOS tend to be younger than participants in typical weight loss programs, and younger women tend to have higher dropout rates generally [17].

Therefore, we aimed to extend the impact and retention of future PCOS diet and lifestyle programs by surveying women with PCOS about their program preferences. Thus, a goal of this research was to provide valuable data that can be used to inform future intervention development for symptom management and disease prevention among women with PCOS.

## Methods

### Setting and Participants

We solicited feedback from women who had been diagnosed with PCOS using an online survey website—Amazon Mechanical Turk. This allowed us to survey women anonymously while still paying them for their participation. We described that, “this survey is for women with Polycystic Ovary Syndrome (PCOS) who might be interested in a diet and lifestyle program to help improve their health… If you don't have PCOS do not continue.” Thus, this was a self-qualified convenience sample.

Participants provided informed consent online. The research was approved by the University of Michigan Institutional Review Board (HUM00127004).

### Measures

Participants used a 7-point Likert scale (1=*not very interested* to 7=*very interested*) to rate their interest in a diet and lifestyle program to achieve each of 9 health outcomes [18] using each of 7 potential program format options [19,20]. They also rated their willingness to make 6 diet and lifestyle changes [21-25] using a 7-point scale (1=*not at all willing* to 7=*very willing*). We measured stress-related eating using the 4-item Eating to Cope subscale of the Palatable Eating Motives Scale (PEMS) [26], which asks people to consider what reasons people give for eating highly palatable foods such as sweets, salty snacks, fast foods, and sugary drinks, rated on a 5-point scale (almost never/never, some of the time, half of the time, most of the time, almost always/always). The 4 prompts include “to forget your worries,” “because it helps you when you feel depressed or nervous,” “to cheer up when you are in a bad mood,” and “to forget about your problems.” We also asked participants 2 open-ended questions about their PCOS and possible programs: “What health concerns do you have related to it?” and “What other aspects of a diet and lifestyle program might be useful?”

### Statistical Analyses

Descriptive data are presented in means, standard deviations, and percentages. We dichotomously classified interest responses of at least 6 out of 7 as “interested” and willingness ratings of at least 6 out of 7 as “willing.” We computed associations between selected predictors (body mass index or BMI, age, education, and PEMS) and continuous ratings of interest and willingness. Given the exploratory nature of the study, we tested Pearson correlations using a conservative criterion of *P*<.01.

## Results

We recruited 197 women with self-identified PCOS. See Table 1 for sample characteristics.

**Table 1:** Baseline characteristics of women with PCOS (N=197).

Characteristics		Values
Age in years, mean (SD)		32.8 (8.1)
**Race/ethnicity** **, n (%)**		
	Asian/Pacific Islander	8 (4.1)
	Black	16 (8.1)
	White	173 (87.8)
	American Indian or Alaskan Native	10 (5.1)
	Latino	22 (11.2)
**Education level, n (%)**		
	Not a college graduate	79 (40.1)
	College graduate	85 (43.1)
	Post graduate education	33 (16.8)
**Total household income, n (%)**		
	Up to US $35,000	63 (32.0)
	US $35,001- US $75,000	99 (50.3)
	Over US $75,000	35 (17.8)
Years since diagnosis of PCOS^a^ years, mean (SD)		6.9 (6.6)
Weight in kg, mean (SD)		81.2 (26.1)
Body Mass Index in kg/m^2^, mean (SD)		29.6 (9.7)
Stress eating,^b^ mean (SD)		2.7 (1.2)

^a^PCOS: polycystic ovary syndrome.

^b^Scores on Eating to Cope subscale of the Palatable Eating Motives Scale (PEMS).

### Potential Program Outcomes

Between 53% and 73% of respondents reported interest in a program that would help them to feel more energetic, feel less anxious and depressed, lose weight, prevent a diabetes onset, regulate menstrual periods, and reduce hirsutism (Table 2).

The older the women were, the more interested they were in increasing their energy and preventing diabetes, but the less they were interested in reducing acne and becoming pregnant. The more overweight the women were, the more interested they were in increasing their energy, reducing their anxiety, losing weight, preventing diabetes, and reducing hirsutism. Greater stress eating was associated with interest in reducing anxiety and depression as well as regulating menstrual periods.

We also asked respondents to report any other health concerns they had about PCOS using an open-ended question. These responses included pain, thinning hair, mood swings, and loss of libido.

**Table 2:** Associations between interest/willingness ratings in potential program features and selected respondent variables.

Variables		Interested^a^ or Willing^b^ participants n (%) N=197	Pearson correlation			
			Age	Education	Body mass index	Stress eating^c^
**Potential psychological outcomes**						
	Feeling more energetic	144 (73.1)	.23*	.07	.27*	.10
	Feeling less anxious	132 (67.0)	.04	.01	.28*	.21*
	Feeling less depressed	124 (62.9)	−.02	−.06	.11	.26*
**Potential physical outcomes**						
	Losing weight	124 (62.9)	.03	−.07	.45*	.16
	Preventing diabetes	116 (58.9)	.17*	.00	.24*	.13
	Regulating menstrual periods	104 (52.8)	−.07	-.09	.12	.23*
	Reducing hirsutism	104 (52.8)	−.04	.04	.23*	.13
	Reducing acne	87 (44.2)	−.27*	.14	.03	.18
	Becoming pregnant	45 (22.8)	−.28*	.07	-.02	.11
**Potential content**						
	Eating a very low-carbohydrate, ketogenic diet	87 (44.2)	.11	.09	.08	.12
	Eating a paleo diet or diet with little processed foods, grains, or dairy	89 (45.2)	.15	.11	.10	.09
	Eating a low–glycemic index diet	87 (44.2)	.21*	.14	.07	.13
	Getting regular physical activity	128 (65.0)	.19*	.10	.04	−.08
	Getting sufficient sleep	160 (81.2)	.09	.07	.14	.09
	Practicing stress-reduction techniques	146 (74.1)	.18	.08	−.02	−.01
**Potential mentoring format**						
	Email	100 (50.8)	.20*	.17	.16	−.01
	In-person	49 (24.9)	−.20*	.09	−.07	−.03
	Phone	37 (18.8)	−.12	.16	−.13	.10
	Video chat	26 (13.2)	−.03	.16	−.16	.06
**Potential lesson format**						
	Online	106 (53.8)	.19*	.13	.21*	.10
	In a mobile application on phone	100 (50.8)	.07	.08	.08	.05
	In-person	37 (18.8)	−.18	.06	−.06	.07

*P<.01.

^a^Response of at least 6 out of 7 on interest scale.

^b^Response of at least 6 out of 7 on willingness scale.

^c^Eating to Cope subscale of the Palatable Eating Motives Scale (PEMS).

### Program Content (Behavior Change Targets)

Between 65% to 81% of respondents reported a willingness to get regular physical activity and sufficient sleep as well as a willingness to practice stress-reduction techniques (Table 2). Only 44%-45% were willing to try each of the 3 diets. Older age was correlated with a willingness to try a low–glycemic index diet and get regular physical activity. Weight, education, and stress eating were not correlated with a willingness to change any of the behaviors.

### Program Format

Between 51% to 54% of respondents were interested in receiving help from a mentor via email, online lessons, or mobile lessons. In contrast, one-fourth or less were interested in receiving mentorship in person, by telephone, or via video chat; and less than one-fifth were interested in in-person lessons. Age was positively associated with interest in being mentored over email and receiving online lessons, and negatively associated with being mentored in person. BMI was positively associated with interest in receiving online lessons. Education and stress eating were not correlated with any program format preferences.

Using an open-ended question, we also asked participants what other aspects of a diet and lifestyle program might be useful. They described other possible features, including getting support from a coach (“just having a person who is there for you”); peers (“I think the support of others going through the same thing could be very meaningful”); or generally anyone supportive (“Constant reassurance would be the most useful tool”). They wanted this support to help keep them accountable to their goals. Participants suggested that the program provide reminders of their goals and rewards for reaching those goals. Some mentioned wanting mobile applications or online or text-based interactions (“Daily goals/reminders through push notifications on a mobile app,” and “I just like accountability without having to talk on the phone or in person. I'm an introvert so I like online message boards, text messages, etc.”) whereas others wanted in-person help (“I would rather just have a checklist of things to do, like a hard copy piece of paper that I can hang on my fridge to help guide my food choices and remind me why those choices are important” and “I like meeting a person in real life because you can see and feel the motivation from them”).

## Discussion

This study aimed to determine which diet and lifestyle program features appeal to women with PCOS. Results suggest that the majority of women prefer programs targeting numerous outcomes, including increasing energy, reducing anxiety and depression, losing weight, lowering diabetes risk, regulating menstruation, and reducing hirsutism. A clear majority were interested in a topical coverage of sleep, stress reduction, and physical activity. There were lower rates of interest in various diets. Finally, about half of the survey respondents expressed interest in email-based mentoring and online or mobile delivery.

Findings could also inform efforts to maximize program appeal to various PCOS subgroups. For example, the younger the women were, the more interested they were in typical concerns of younger women, including reducing acne and increasing fertility. Further, the higher a woman’s BMI, the more she preferred a program to help with weight loss, improved energy, and reduced anxiety.

To our knowledge, this is the first study to examine program attitudes and preferences in this population. However, a primary study limitation is the online recruitment of a convenience sample. This may at least partially explain reported preferences for online or mobile programs and email-based mentoring. Additionally, self-report biases such as social desirability may have affected respondents’ ratings of interest and willingness. Nonetheless, future online and mobile diet and lifestyle programs may be able to capitalize on this information to better target this population’s expressed preferences.

Overall, we hope that this research can help inform future diet and lifestyle programs for women with PCOS. Ideally, this will enable the programs to have fewer participants who drop out of the programs and more satisfied and adherent participants.

## Abbreviations

BMI: body mass index
PCOS: polycystic ovary syndrome
PEMS: Palatable Eating Motives Scale
